# Effective data filtering is prerequisite for robust microbial association network construction

**DOI:** 10.3389/fmicb.2022.1016947

**Published:** 2022-10-04

**Authors:** Mengqi Wang, Qichao Tu

**Affiliations:** ^1^Institute of Marine Science and Technology, Shandong University, Qingdao, China; ^2^Joint Lab for Ocean Research and Education at Dalhousie University, Shandong University, Qingdao, China; ^3^Joint Lab for Ocean Research and Education at Dalhousie University, Xiamen University, Qingdao, China; ^4^Southern Marine Science and Engineering Guangdong Laboratory (Zhuhai), Guangzhou, China

**Keywords:** microbial community, zero values, data filtering, correlation inference, association networks

## Abstract

Microorganisms do not exist as individual population in the environment. Rather, they form complex assemblages that perform essential ecosystem functions and maintain ecosystem stability. Besides the diversity and composition of microbial communities, deciphering their potential interactions in the form of association networks has attracted many microbiologists and ecologists. Much effort has been made toward the methodological development for constructing microbial association networks. However, microbial profiles suffer dramatically from zero values, which hamper accurate association network construction. In this study, we investigated the effects of zero-value issues associated with microbial association network construction. Using the TARA Oceans microbial profile as an example, different zero-value-treatment approaches were comparatively investigated using different correlation methods. The results suggested dramatic variations of correlation coefficient values for differently treated microbial profiles. Most specifically, correlation coefficients among less frequent microbial taxa were more affected, whichever method was used. Negative correlation coefficients were more problematic and sensitive to network construction, as many of them were inferred from low-overlapped microbial taxa. Consequently, microbial association networks were greatly differed. Among various approaches, we recommend sequential calculation of correlation coefficients for microbial taxa pairs by excluding paired zero values. Filling missing values with pseudo-values is not recommended. As microbial association network analyses have become a widely used technique in the field of microbial ecology and environmental science, we urge cautions be made to critically consider the zero-value issues in microbial data.

## Introduction

Rather than simple accumulation of individual populations, microorganisms in natural ecosystems may interact with each other and form complex assemblages, thereby execute essential ecosystem functions (e.g., biogeochemical cycling of various nutrients) and maintain ecosystem stability (Fuhrman et al., 2015). Therefore, in addition to the community structure and composition, their interactive relationships shall also be critically considered for better understanding the roles that microbial communities play in both natural and artificial ecosystems. However, due to the complexity and not yet cultured majority of microbial taxa, direct observation of such complex relationships is generally not feasible (Trosvik et al., 2010; Berry and Widder, 2014). Recent advances in high throughput metagenomic technologies make it possible to capture genomic information for thousands of microbial taxa in a single experiment (van Dijk et al., 2014; Slatko et al., 2018). Since then, multiple statistical approaches, such as SparCC (Friedman and Alm, 2012), MENA (Deng et al., 2012), LSA (Xia et al., 2013), CoNet (Faust and Raes, 2016), and SPIEC-EASI (Kurtz et al., 2015), have been developed to infer the complex relationship among microbial taxa based on the (relative) abundance profiles, in the form of association networks.

Notably, these different approaches mainly focus on different statistical methods for pairwise correlation coefficients calculation and network construction. For example, SparCC focuses on correlation inference of compositional data, and uses Aitchison’s variance of log-ratios to solve ingredient problems (Friedman and Alm, 2012). MENA uses Random Matrix Theory (RMT) to identify a cutoff for constructing microbial association networks, by determining the transition point of nearest-neighbor spacing distribution of eigenvalues from Gaussian (random) to Poisson (non-random) distribution (Deng et al., 2012). LSA breaks down the global molecular similarity as local similarity at each grid point surrounding the molecules and is efficient to calculate statistical significance for pairwise local similarity analysis, making possible all-to-all local association studies otherwise prohibitive. The LSA approach is commonly used to construct association network from time series data (Xia et al., 2013). CoNet offers ensemble-based network construction, by combining a number of different correlations (Pearson, Spearman, and Kendall), similarities (Mutual information) or dissimilarities (Bray-Curtis and Kullback-Leibler) to improve the accuracy of the strength of the associations between objects (Faust and Raes, 2016). SPIEC-EASI is a computational framework that includes statistical methods for the inference of microbial ecological interactions from 16S rRNA gene sequencing datasets, and is equipped with a sophisticated synthetic microbiome data generator with controllable underlying species interaction topology (Kurtz et al., 2015).

Microbial communities in natural ecosystems are usually consisted by a few dozens of abundant and occasional taxa, and a long “tail” of rare taxa (Logares et al., 2014). As a result, zero values are especially common in microbial community profiles, especially for occasional and rare taxa, suggesting that these microbial taxa could be either biologically absent or not detected by the current sequencing effort. Consequently, inadequate considering the issues associated with zero values may lead to problematic microbial association network construction. However, current studies constructing microbial association networks generally input the (log-transformed) (relative) abundance profiles to the abovementioned approaches and do not specifically consider the situation of zero values in microbial profiles.

In this study, we aimed to investigate the issues associated with correlation coefficient inference and association network construction caused by zero values in microbial profiles. Multiple situations for dealing with zero values in microbial profiles were considered and comparatively evaluated for association network construction. The results demonstrated that different data filtering methods for zero values dramatically interfere microbial association network construction. Different data filtering methods may exert as strong variation in correlation coefficient values as network correlation inference methods. Such issues were especially critical for negative association inference. Therefore, we urge that the common zero values in microbial profiles be thoroughly considered for robust microbial association network construction.

## Materials and methods

### Methodological framework

**FIGURE 1:**
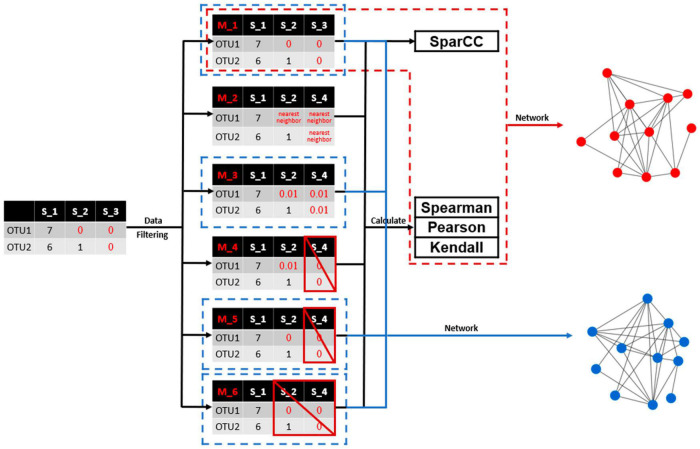
A flowchart illustrating the overall methodology workflow of this study. A typical microbial profile containing large number of missing values was selected as the example dataset. Different data filtering methods were then applied to process the missing values associated with the microbial profile. Commonly used correlation coefficient calculation methods including the SparCC, Spearman, Pearson, and Kendall were applied to calculate the pairwise correlation among microbial taxa. Association networks were then constructed and comparatively analyzed.

A framework was presented to illustrate the overall methodology flow of this study (Figure 1). A typical microbial profile containing large number of zero values was selected as the example dataset. Different data filtering methods were then applied to process the zero values associated with the microbial profile. The commonly used correlation coefficient calculation methods including the Spearman, Pearson and Kendall were applied to calculate the pairwise correlation among microbial taxa. The SparCC method was also employed to construct microbial association networks based on the microbial profile containing large number of zero values. Association networks were then constructed with identical cutoffs and comparatively analyzed, in order to evaluate the effects of different data filtering methods.

### The TARA oceans microbial profile

We first tried to generate synthetic microbial profiles using models like generalized Lotka–Volterra (gLV) model (Hernández-Bermejo and Fairén, 1997). However, effective dataset containing comparable zero-values to experimentally generated datasets cannot be generated. Due to the lack of standard microbial profiles with zero values and known relationships, we used a real dataset, the TARA Oceans metagenome, to illustrate the effects of different data filtering methods on microbial correlation coefficient calculation. The TARA Oceans metagenomic dataset was selected because it is a representative dataset covering 139 microbial communities in the largest fluid ecosystem in the Earth’s biosphere. Zero-values are common in this dataset, satisfying the requirement of this study. The miTAG based microbial taxonomic profile was downloaded from http://ocean-microbiome.embl.de/companion.html. The row annotated as unclassified taxa was first removed from the microbial profile for its representing an aggregation of many unknown microbial OTUs. The profile was then rarefied to a same sequencing depth (39,410 reads per sample). In the profile, a total of 35,650 OTUs were found. The absolute value of OTU abundance after rarefaction was retained for correlation coefficient calculation. In addition to direct using abundance data for correlation calculation, several studies suggest that microbial profiles are compositional (Jackson, 1997; Friedman and Alm, 2012). Therefore, in addition to rarefied abundance data, the microbial profile was also centered log transformed and subject to correlation calculation. The R package “compositions” was used for centered log transformation (van den Boogaart and Tolosana-Delgado, 2008).

### Data filtering and correlation coefficient calculation

Six different data filtering methods were employed to deal with zero values (Figure 1). The first method is to treat zero values (i.e., NA values) as 0. The second method is to use a nearest neighbor algorithm instead of zero values (Cover and Hart, 1967), by which zero values were replaced by inferred expectations based on observed data. The R package “DMwR2” was used for nearest neighbor employment (Torgo, 2016). The third method is to use 0.01 instead of zero values, which is the default value recommended by the MENA pipeline (Deng et al., 2012). The fourth method is to exclude the samples from correlation calculation in which pairwise zero values are detected and the unpaired zero values are replaced with 0.01. The fifth method is similar to the fourth method but unpaired zero values are kept as is. The sixth method is to exclude all samples from correlation calculation as long as paired or unpaired zero values are observed.

Afterward, correlation methods including Spearman, Pearson, and Kendall, which reflect the direction and degree of the change trend between two variables, were employed for correlation coefficient calculation for the treated microbial profiles. Correlation coefficient values were sequentially calculated for each pair of OTUs meeting the minimum requirement (i.e., larger than 30 paired valid observations). Since the data filtering methods are not applicable to SparCC, only the raw microbial profile containing zero values was subject to correlation calculation and network construction by SparCC. Here, the python package SparCC3^1^ was used, with the iteration value set to 20. Since SPIEC-EASI is based on penalized estimators, the edge weights are not directly comparable to SparCC and Pearson/Spearman correlation coefficients, this method was not investigated here. In some cases (e.g., methods 4, 5, and 6), OTU pairs that did not meet the minimum requirement (i.e., larger than 30 paired valid observations) for correlation calculation were excluded. The calculated correlation coefficients by different data filtering methods and correlation methods were comparatively analyzed. For better illustration, a total of 10,000 OTU pairs were randomly selected. The associated R code and documents for correlation calculation are available at https://github.com/qichao1984/DataFiltering.

### Microbial association network construction

In many approaches, an empirical cutoff is selected for association network construction, except the MENA pipeline. Here, for evaluation purpose, a correlation cutoff with absolute value larger than 0.6 and *P*-value smaller than 0.001 were chosen for network construction. As in many other studies, we focus on the patterns of association networks and do not expect strong effects of this cutoff on the results and conclusions. In addition, Random Matrix Theory (RMT) was also employed via the iNAP pipeline (Feng et al., 2022) to identify thresholds for constructing highly confident microbial ecological networks, together with the *P*-value cutoff of 0.001 (Zhou et al., 2010; Feng et al., 2022). Two types of association networks, including co-occurrence and co-exclusive networks, were constructed and analyzed. In the co-occurrence network, only positive associations were included, whereas, in the co-exclusive network, only negative associations were included. Microbial association networks were constructed for microbial profiles subject to different data filtering methods and correlation methods. The structural and topological differences among the constructed networks were comparatively inspected.

To better illustrate how microbial association networks differ from each other, subnetworks were also constructed targeting the first neighbors of top ten most connected nodes identified in the fifth data filtering methods. In addition, consensus networks were constructed based on differently treated microbial profiles and different correlation methods, in order to see the variations caused by data filtering methods and correlation methods. The constructed networks were visualized and analyzed by the Cytoscape software (Shannon et al., 2003).

## Results

### Zero values were rarely critically considered in microbial association network constructions

**FIGURE 2:**
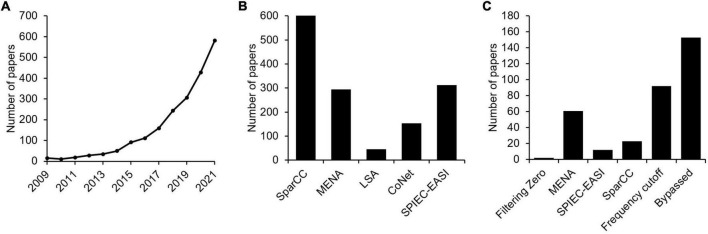
The current situation of implementing microbial association network analyses in microbial ecological and environmental studies by analyzing published literatures. **(A)** Using the keywords “microbial ecological network” and “microbial co-occurrence network,” the number of papers published in the NCBI PubMed database in the past 13 years was analyzed. **(B)** Citations of different microbial association network construction methods. **(C)** The number of papers whether missing values were considered when constructing microbial association networks.

We first investigated the current situation of implementing microbial association network analyses in microbial ecological and environmental studies by analyzing published literatures. Using the keywords “microbial ecological network” and “microbial co-occurrence network,” it was found that the number of published studies in the NCBI PubMed database increased dramatically over the past 13 years. Specifically, the number of indexed literatures per year achieved to ∼581 in December 2021, whereas the number was only ∼15 in 2009 (Figure 2A). This means that, as a recently developed technology in the metagenomic era, microbial association network analyses have become a routine approach in microbial ecological and environmental studies. Among these studies, SparCC is the method with most citations, followed by SPIEC-EASI and MENA (Figure 2B).

Although commonly applied, it should be pointed out that issues associated with zero values have rarely been critically considered in many studies. For computational purposes, only minimal treatments have been employed by different association network construction approaches (Table 1). For instance, methods like SparCC and SPIEC-EASI assign a pseudo-count of 1 to zero values, to avoid log transformation of zero value (Friedman and Alm, 2012; Kurtz et al., 2015). The MENA pipeline (Deng et al., 2012), which mainly employs Pearson correlation coefficient and Spearman’s rank order correlation for association inference, fills a pseudo-value of 0.01 to zero values by default, with the option of user defined values available. A common agreement reached by computational microbial ecologists is that OTUs showing up in only a few samples should be excluded from correlation analyses, as they may lead to false association inferences (Huse et al., 2010; Haas et al., 2011; Reshef et al., 2011; Degnan and Ochman, 2012; Faust, 2021). More critically, literature analysis suggested that no specific attention was paid to zero value issues by many studies (Figure 2C), as judged by whether zero values are specifically mentioned in the methods section, though it is possible that some studies do have considered zero values but did not mention it in the paper. Ignoring such issues may impose severe consequences to the conclusions drawn in the studies, such as inaccurate (false positive and false negative) inference of microbial association relationships. It is therefore of necessity to investigate how zero values may affect microbial association network inference.

**TABLE 1:** Characteristic and zero-value-treatment methods of the commonly used microbial association network construction methods.

Method	Characteristic	Zero value treatment
SparCC (6)	SparCC focuses on correlation inference of compositional data, and uses Aitchison’s variance of log-ratios to solve ingredient problems. It could be used on any genomic survey data that has low diversity.	Add a pseudo-value of 1 to each element.
MENA (7)	MENA uses Random Matrix Theory (RMT) to identify a cutoff for constructing microbial association networks, by determining the transition point of nearest-neighbor spacing distribution of eigenvalues from Gaussian (random) to Poisson (non-random) distribution.	By default, unpaired zero values were filled by a pseudo-value of 0.01. More user defined options are also available.
LSA (8)	LSA breaks down the global molecular similarity as local similarity at each grid point surrounding the molecules and is efficient to calculate the statistical significance for pairwise local similarity analysis, making possible all-to-all local association studies otherwise prohibitive. It is commonly used to construct association network from time series data.	No mention of the treatment of zero values.
CoNet (9)	CoNet offers ensemble-based network construction, by combining a number of different correlations (Pearson, Spearman, and Kendall), similarities (Mutual information) or dissimilarities (Bray-Curtis and Kullback-Leibler) to improve the accuracy of the strength of the associations between objects.	Omitting sample pairs with zero values from the association strength calculation.
SPIEC-EASI (10)	SPIEC-EASI is a computational framework that includes statistical methods for the inference of microbial ecological interactions from 16S rRNA gene sequencing datasets. A sophisticated synthetic microbiome data generator with controllable underlying species interaction topology is also equipped.	Add a pseudo-value of 1 to zero values.

### Issues associated with microbial community profiles

Using the TARA Oceans microbiome data as an example, we first illustrated the typical data structure of microbial profiles and how different data filtering methods would affect them. Although the following issues may have already been recognized by the community, we believe they still deserve to be further emphasized here.

**FIGURE 3:**
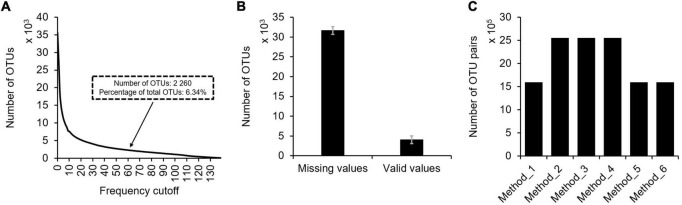
The situation of missing values in typical microbial profiles. Using the TARA Oceans miTAG microbial profile as an example, the following information was shown: **(A)** The number of OTUs under different frequency cutoff. With 60 samples as the frequency cutoff, the number of detected OTUs was 2,260, accounting for 6.34% of all OTUs. **(B)** The average number of missing values and valid values present in each sample with standard error. **(C)** The number of OTU pairs suitable for correlation analyses using different data filtering methods.

First, zero values are common in microbial profiles. As previously reported, microbial communities are usually composed by a small number of abundant taxa and a long “tail” of rare taxa (Logares et al., 2014), meaning the existence of many zero values in microbial profiles. Although thousands to tens of thousands microbial taxa could be identified in a routine study, only a small portion of them (6.34%) were present in multiple samples (e.g., 60 samples) (Figure 3A). In each sample of the profile, more than 88.69% observations are zero (Figure 3B). The meaning of these zero values is uncertain that they could either be biologically absent from the sample or technically not detected. In addition, random subsampling of microbial profiles to an equal sequencing depth may also result in zero values for rare taxa.

Second, not all OTUs shall be subject to correlation analyses for association network construction. Correlation coefficient between two OTUs can be calculated as long as they are present in three or more samples. And confident estimation of correlation coefficients between two OTUs requires more valid observations. However, the number of OTUs decreases with the number of samples they show up. For instance, 2 260 (6.34%) OTUs were present in ≥60 samples in the TARA Oceans expedition, though a total of 35,650 annotated OTUs were identified (Figure 3A). To our best knowledge, no standard or consensus criteria is available to define a sample number cutoff for selecting OTUs. Empirically, the MENA pipeline recommended a cutoff of showing up in 50% samples to select OTUs for correlation analyses. However, large scale studies (both spatial and temporal, e.g., studies encompassing hundreds of samples at the continent or global scale) may invalidate such empirical recommendations as very few microbial taxa may show up in ≥50% samples.

Third, treating zero values differently may lead to dramatically differed input microbial profiles for correlation analyses, further affecting association network inference. Multiple data filtering methods are available (Figure 1). Taking the TARA Oceans microbial profile containing OTUs showing up in ≥60 samples for example, the number of OTU pairs suitable for correlation analyses varied dramatically for different data filtering methods (Figure 3C).

### Data filtering methods interfere microbial correlation inference

We then investigated how different data filtering methods may affect microbial correlation inference. As multiple microbial correlation inference methods are available, here only those more commonly used approaches were investigated, including the Pearson correlation coefficient, Spearman’s rank order correlation, and the non-parametric Kendall rank correlation. The correlation coefficient values returned by the SparCC approach was also assessed. These are also the correlation methods used by most microbial association network construction methods. Correlation methods such as Bray-Curtis dissimilarity, Mutual Information, and Maximal Information Coefficient were not considered here due to their returned values always ≥0, failing to encompass negative correlations in microbial association relationships in the same way and criteria as other methods did.

**FIGURE 4:**
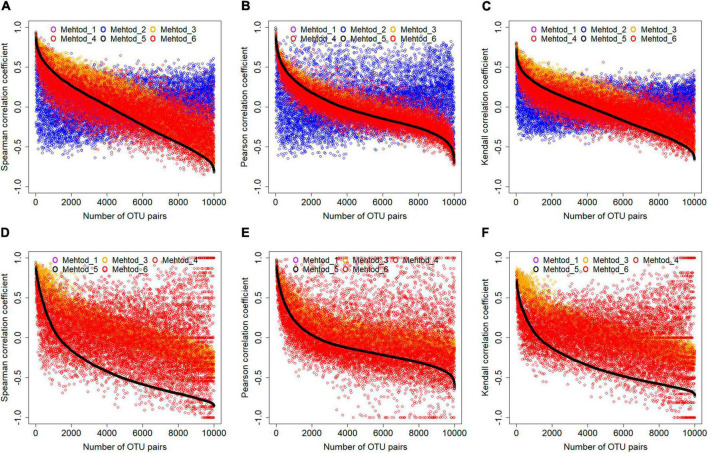
The effects of data filtering methods on correlation coefficient calculation. Two types of datasets, including OTUs showing up in ≥60 samples **(A–C)** and OTUs showing up in ≥30 samples and ≤60 samples **(D–F)** were analyzed. Three different correlation calculation methods were evaluated, including the Spearman, Pearson, and Kendall. For better visualization, correlation coefficient values of 10,000 randomly selected OTU pairs were plotted. For the less frequent dataset **(D–F)**, nearest neighbor algorithm was not applicable.

First, treating zero values differently resulted in dramatically different correlation coefficients. Here, microbial OTUs showing up in ≥60 samples were extracted and subjected to data filtering by all six methods. Correlation coefficients among different OTUs were calculated for the filtered microbial profiles. Using correlation coefficients among OTUs with paired abundance values (i.e., method 5) as standard, it was found that different data filtering methods resulted in different correlation coefficient values, no matter which correlation method was used (Figures 4A–C). Specifically, the correlation coefficient values for microbial profile whose zero values were filled by nearest neighbor algorithm showed the strongest variations from those by other methods. Correlation coefficient values for microbial profile whose zero values were filled with 0.01, which is the default approach used by the MENA pipeline, were much higher than the standard. Notably, correlation coefficient values were also sensitive to correlation methods. Pearson correlation coefficient seemed to be more robust than Spearman’s rank correlation and Kendall’s rank correlation that correlation coefficient values were more centered to the standard for abundance-based microbial profiles with different treatment of zero data, except that filled by the nearest-neighbor algorithm.

Second, correlation inference for less frequent microbial taxa was more affected. Microbial profiles consisted by OTUs showing up in ≥30 samples and ≤60 samples were also extracted and evaluated. In the nearest neighbor algorithm, missing values are inferred from its neighboring samples around. Therefore, missing values could not be inferred using the nearest neighbor algorithm for less frequent microbial taxa. As a result, comparing to the microbial profiles consisted of OTUs showing up in ≥60 samples, the correlation coefficient values for less frequent OTUs were much more sparsely distributed, no matter which correlation calculation method was used (Figures 4D–F). This suggested that cautions should be taken for microbial association network construction based on less frequent OTUs, either for choosing correlation calculation or data filtering methods.

Third, negative correlations could be more problematic. We also investigated how different data filtering methods affected positive and negative inference of microbial associations (Supplementary Figure 1). The number of strong positive and negative microbial associations (| corr| > 0.6) greatly varied for different data filtering methods. This was especially critical for microbial profiles whose zero values were replaced by the nearest neighbor algorithm and subject to correlation calculation by Spearman and Kendall correlation methods. For positive inference, the majority of strong positive correlation were identified among microbial taxa whose overlapped observations were larger than 60 (Supplementary Figures 1A–C). For negative inference, a large portion of strong associations were identified among microbial taxa with fewer overlapped observations, especially for those calculated by Spearman and Kendal correlation methods. Negative correlation coefficients were lowly detected using Pearson and Kendall correlation methods (Supplementary Figures 1D–F). Such results suggested that negative association inference for microbial association networks were more strongly affected by data filtering methods. The results also provided potential clues explaining why negative associations were rarely found in many studies.

Fourth, log-transformation of the abundance data improved the centrality of the distribution of correlation coefficient values to the standard. Several studies suggested that the abundance data of microbial profiles is compositional (Jackson, 1997; Friedman and Alm, 2012). A common approach to treat such data is log transformation. Here, the abundance profiles of microbial communities were centered log transformed and subjected to correlation coefficient calculation. As a result, comparing to untransformed microbial profiles, correlation coefficient values were overall more centralized to the standard. Such result was consistent across different correlation methods and different frequency cutoffs of microbial profiles (Supplementary Figure 2). Again, the correlation coefficient values for microbial profiles whose zero values were filled by nearest neighbor were always sparsely distributed from the standard. This suggested that the nearest neighbor algorithm seemed not a good way for microbial community data preprocessing, though this method has been widely applied in genomic studies (Parry et al., 2010; Belka et al., 2018; Tjarnberg et al., 2021; Xie et al., 2021).

Finally, correlation coefficient calculation method like SparCC may also be affected. Since data filtering methods are not applicable to SparCC, which is one of the representative methods that treat microbial community data as sparse, we compared the correlation coefficient values calculated by Spearman, Pearson, and Kendall correlation methods with that by SparCC (Supplementary Figure 3). As a result, the correlation coefficient values between SparCC and other methods were all strongly correlated (R^2^ > 0.95, *P* < 2.2e-16). This suggested that the same issue encountered by other correlation methods also tended to exist in methods like SparCC.

### Microbial association networks dramatically differed

**FIGURE 5:**
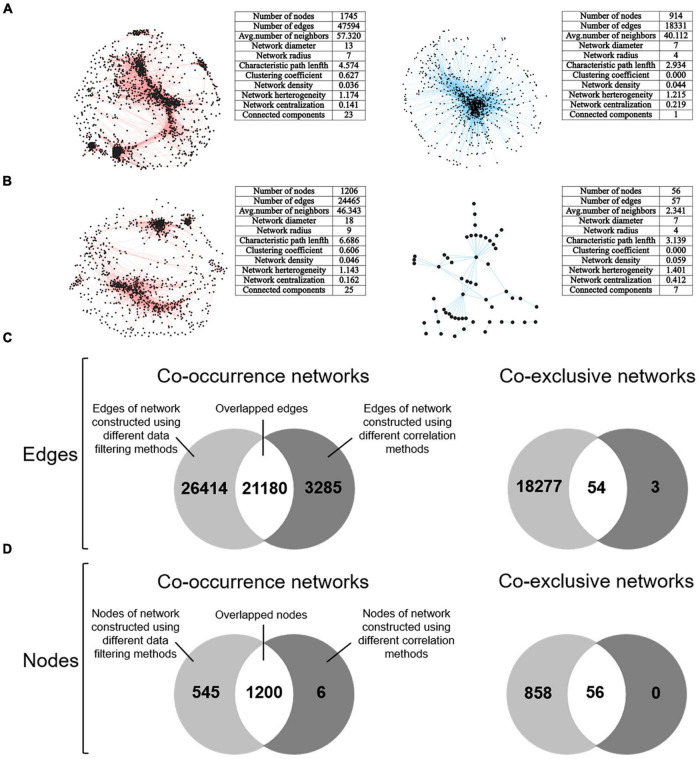
Consensus networks representing microbial co-occurrence and co-exclusive associations were constructed and comparatively analyzed. **(A)** Consensus co-occurrence and co-exclusive networks constructed by extracting the overlapped nodes and edges based on networks constructed using the first, third, fifth, and sixth data filtering method (refer to Figure 1). The Spearman’s rank order correlation coefficient was used for network construction. **(B)** Consensus co-occurrence and co-exclusive networks constructed by extracting the overlapped nodes and edges based on networks constructed using different correlation methods, including Spearman, Pearson, Kendall, and SparCC. The first data filtering method (filling missing values with zero) was used here. **(C)** Overlapped edges between the consensus networks of different data filtering methods and correlation methods. **(D)** Overlapped nodes between the consensus networks of different data filtering methods and correlation methods. In panels **(A,B)**, networks with red edges represent co-occurrence networks, and networks with blue edges represent co-exclusive networks.

Finally, microbial association networks were constructed and comparatively analyzed. Correlation coefficient cutoffs of 0.6 and –0.6 (*P* < 0.001) were respectively used to identify positive and negative associations among different microbial taxa. Using the microbial association networks constructed based on Spearman correlation coefficient as examples, dramatically differed networks were obtained, both topologically and in content (Supplementary Figure 4). For both co-occurrence and co-exclusive networks, distinct network structures were observed for microbial profiles subject to different data filtering methods. Such distinct network topology resulted in distinct network parameters such as the number of connected nodes/edges, average number of neighbors, network diameter, network radius, characteristic path length, clustering coefficient, network density, network heterogeneity, network centralization, and connected components. We then extracted the subnetworks of top ten most connected nodes (Supplementary Figure 5), which is the commonly used approach to investigate the network topology of the most connected microbial taxa. More strikingly, the extracted subnetworks suggested that different data filtering methods resulted in different clues in finding out the most “important” microbial taxa in the ecosystem. Further, we constructed and compared the consensus networks by extracting the overlapped nodes and edges in the networks constructed using different data filtering methods and correlation methods (Figure 5). As a result, great difference among different data filtering methods could be found. Highly dissimilar consensus network was observed for different data filtering methods and different correlation methods.

In addition, the iNAP pipeline was also employed to determine the cutoffs for network construction based on RMT method. Similar analyses were then carried out (Supplementary Figures 6–8). In general, consistent result was observed that microbial association networks highly differed for different data filtering methods. However, as the cutoffs determined by RMT were much larger than 0.6, the constructed networks were strongly altered with much fewer nodes and links, especially for co-exclusive networks. Such scenario was expected considering the large cutoffs used for network construction. Based on the above results, we demonstrated that data filtering was also a critical issue as the selection of correlation methods.

## Discussion

As a sophisticated computational approach developed about 10 years ago (Weiss et al., 2016; Röttjers and Faust, 2018), microbial association network analyses have been widely performed in microbial ecology and associated fields to reveal the potential relationships among microbial taxa (Barberán et al., 2012; de Vries et al., 2018; Guo et al., 2022), as well as those between microbes and environmental factors (Chaffron et al., 2010; Lima-Mendez et al., 2015). With proper experimental design and data interpretation, interesting studies that cannot be achieved conventionally have been carried out, such as accurate predicting the biotic interactions among microbes (Barzel and Barabási, 2013; Feizi et al., 2013; Lv et al., 2019), identifying microbial taxa belonging to the same ecological niche (Ma et al., 2020), and revealing the community diversity from the angle of microbial interconnected relationships (Ma et al., 2016; Tu et al., 2020). Foreseeably, the approach will be much more intensively employed by researchers from various fields to address important biological, ecological and environmental questions.

Similar to other approaches, microbial association network analyses also confront a series of caveats and challenges, from methodology to interpretation of the results. A series of issues have been recently discussed by Faust (2021) and Goberna and Verdú (2022). Among these, the issue of zero values is also emphasized in context with rare taxa, for which the majority values are filled with zero. However, the issue of zero values is not only associated with rare, but also abundant and occasional taxa, though with lesser extent. Most correlation coefficient measurements are not designed to consider zero values (i.e., zeros), except few such as Bray-Curtis dissimilarity, which automatically ignores matching zeros between microbial taxa pairs (Faust, 2021). However, dissimilarity values of Bray-Curtis range from 0 to 1, making it hard to define negative correlations in the same manner as other methods.

Current effort mainly focuses on methodology development and novel applications of the approach, leaving the zero-value issue less attended. In previous attempts, researchers generate simulated datasets to validate the accuracy and robustness of the network construction methods they developed (Friedman and Alm, 2012; Kurtz et al., 2015; Yang et al., 2021). However, to our best knowledge, the complex issue of zero values in microbial profiles cannot be well-simulated. Recently, Cougoul et al. (2019) proposed a framework to compute the upper bounds beyond which associations become less meaningful. This study demonstrated that the zero-value issue could be one of the most critical issues in microbial association network construction. As a computational approach, microbial association network analyses also comply the classic “garbage in, garbage out” bioinformatics principle, in which problematic outputs are given by problematic input data (Mellin, 1957; Babbage, 1994). From this point of view, proper data input could be even more important than the choice of correlation methods and network construction methods. This was also confirmed in this study that different data filtering methods resulted in dramatically varied correlation coefficient values using the same correlation method. Different data filtering methods may exert as strong variation on correlation coefficient values as different correlation methods.

Integrating all the results obtained in this study, we propose to critically consider the following caveats for more confident and robust microbial association network construction. First, the frequent zero values in microbial profiles may cause highly differed correlation inference in microbial association network construction. In general, paired valid values are expected to generate robust inference of both positive and negative associations, whereas unpaired valid values could be of great importance for negative associations. Not only shall attention be paid to rare taxa (Faust, 2021), but also abundant and occasional taxa are affected. Second, filling zero values with pseudo values is not recommended. Biased correlation values are observed for microbial profiles filled with pseudo values. The commonly used approaches in the genomic era, e.g., filling zero values using nearest neighbor algorithm (Torgo, 2016; Tjarnberg et al., 2021; Dann et al., 2022), do not seem to be effective in metagenomics. Third, rare and abundant taxa shall be differently treated prior to network construction. For rare taxa with less statistical power, either prevalence removal or forbidding computing correlation coefficients among rare taxa with large number of matching zeros shall be considered, though it may ignore valuable information carried by them (McMurdie and Holmes, 2014; Faust, 2021). Notably, cautions should be made to avoid altered relative abundance due to the removal of rare taxa. For correlation inference among abundant taxa, paired zeros shall be excluded for their biologically insignificance and reducing the statistical power of confident observations. As such, sequential correlation inference between taxa pairs is recommended to avoid miscellaneous issues associated with correlation inference based on whole microbial profile. Many R functions for correlation calculation usually treat and include zero values as is. Fourth, different data filtering methods result in dramatically different microbial association networks. It is therefore necessary for researchers to thoroughly consider the zero values associated with microbial profiles and choose the appropriate one for correlation calculation in network construction. This is an especially critical issue for negative association inference. Whether and how data filtering is carried out for association network construction shall be clearly stated in the manuscripts.

In conclusion, this study investigated the zero-value issue associated with microbial association network construction, which is an issue rarely critically considered in many of the current studies. Different data filtering methods resulted in highly differed association inference for network constructions, especially for negative association inference. As microbial association network approaches are being widely applied in various fields, even to infer relationships among microbial taxa (Barberán et al., 2012; de Vries et al., 2018; Guo et al., 2022), environmental factors (Chaffron et al., 2010; Lima-Mendez et al., 2015), and organic molecules (Li et al., 2018, 2019; Zhao et al., 2019), we urge this issue be critically considered in future studies. Whether and how zero values are treated shall be clearly stated in each study.

## Data availability statement

The original contributions presented in this study are included in the article/Supplementary material, further inquiries can be directed to the corresponding author.

## Author contributions

MW: conceptualization, software, investigation, formal analysis, and writing – original draft. QT: conceptualization, funding acquisition, resources, supervision, and writing – review and editing. Both authors contributed to the article and approved the submitted version.

## Footnotes

1
https://github.com/JCSzamosi/SparCC3
