# Supplementary material for: Effective data filtering is prerequisite for robust microbial association network construction
Source: Front Microbiol. 2022 Oct 4;13:1016947. doi: 10.3389/fmicb.2022.1016947 (PMC9577025; doi:10.3389/fmicb.2022.1016947)
Supplement: Supplementary file 1 [file Data_Sheet_1.pdf]

Effective data filtering is prerequisite for robust microbial association network construction

Mengqi Wang[1], Qichao Tu[1,2,3*]

[1] Institute of Marine Science and Technology, Shandong University, Qingdao, China

[2] Joint Lab for Ocean Research and Education at Dalhousie University, Shandong University and Xiamen University, Qingdao, China

[3] Southern Marine Science and Engineering Guangdong Laboratory (Zhuhai), Guangzhou, China

[*] Correspondence should be addressed to tuqichao@sdu.edu.cn

Running title: Data filtering interferes microbial association networks

Supplementary figures

Supplementary Figure 1. The effects of different data filtering methods on positive and negative inference of microbial associations.

Supplementary Figure 2. The effects of data filtering methods on correlation inference using centered log transformed microbial profiles.

Supplementary Figure 3. Comparison of the correlation coefficient values calculated by Spearman, Pearson, and Kendall correlation methods with that by SparCC.

Supplementary Figure 4. The microbial co-occurrence and co-exclusive networks were based on Spearman correlation coefficient, using different data filtering methods.

Supplementary Figure 5. The microbial co-occurrence and co-exclusive subnetworks of first neighbors of top ten most connected nodes were identified in the fifth data filtering method based on Spearman correlation coefficient, using different data filtering methods.

Supplementary Figure 6. The microbial co-occurrence and co-exclusive networks were based on Spearman correlation coefficient, using different data filtering methods. The RMT method was used to determine correlation coefficient cutoffs via the iNAP pipeline.

Supplementary Figure 7. The microbial co-occurrence and co-exclusive subnetworks of first neighbors of top ten most connected nodes were identified in the fifth data filtering method based on Spearman correlation coefficient, using different data filtering methods. The RMT method was used to determine correlation coefficient cutoffs via the iNAP pipeline.

Supplementary Figure 8. Consensus networks were constructed and comparatively analyzed by applying the iNAP pipeline to determine cutoffs for network construction based on RMT method.

Supplementary Figures

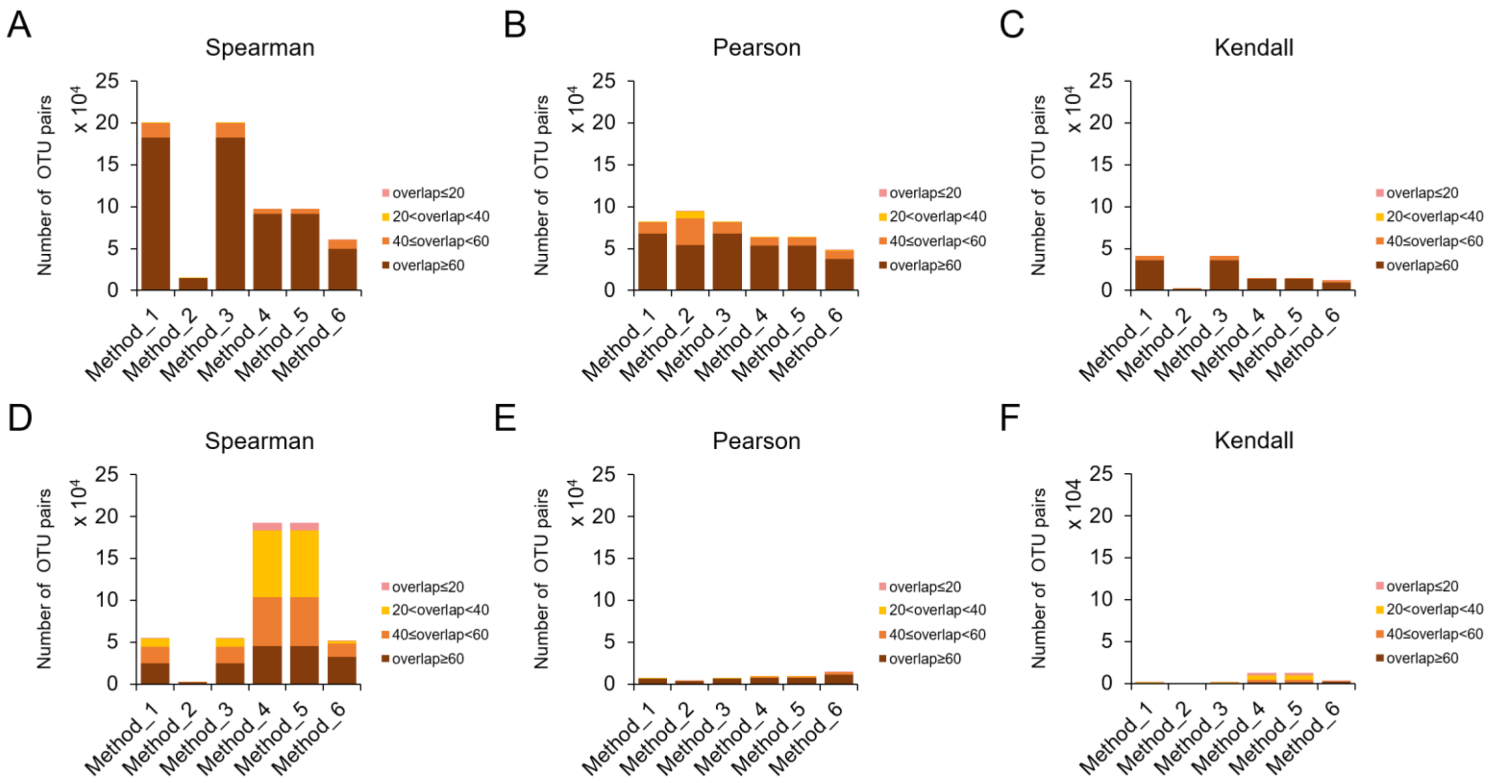

Supplementary Figure 1. The effects of different data filtering methods on positive (A-C) and negative (D-F) inference of microbial associations among OTUs showing up in ≥ 60 samples. Three different correlation methods were employed here, including the Spearman (A and D), Pearson (B and E), and Kendall (C and F).

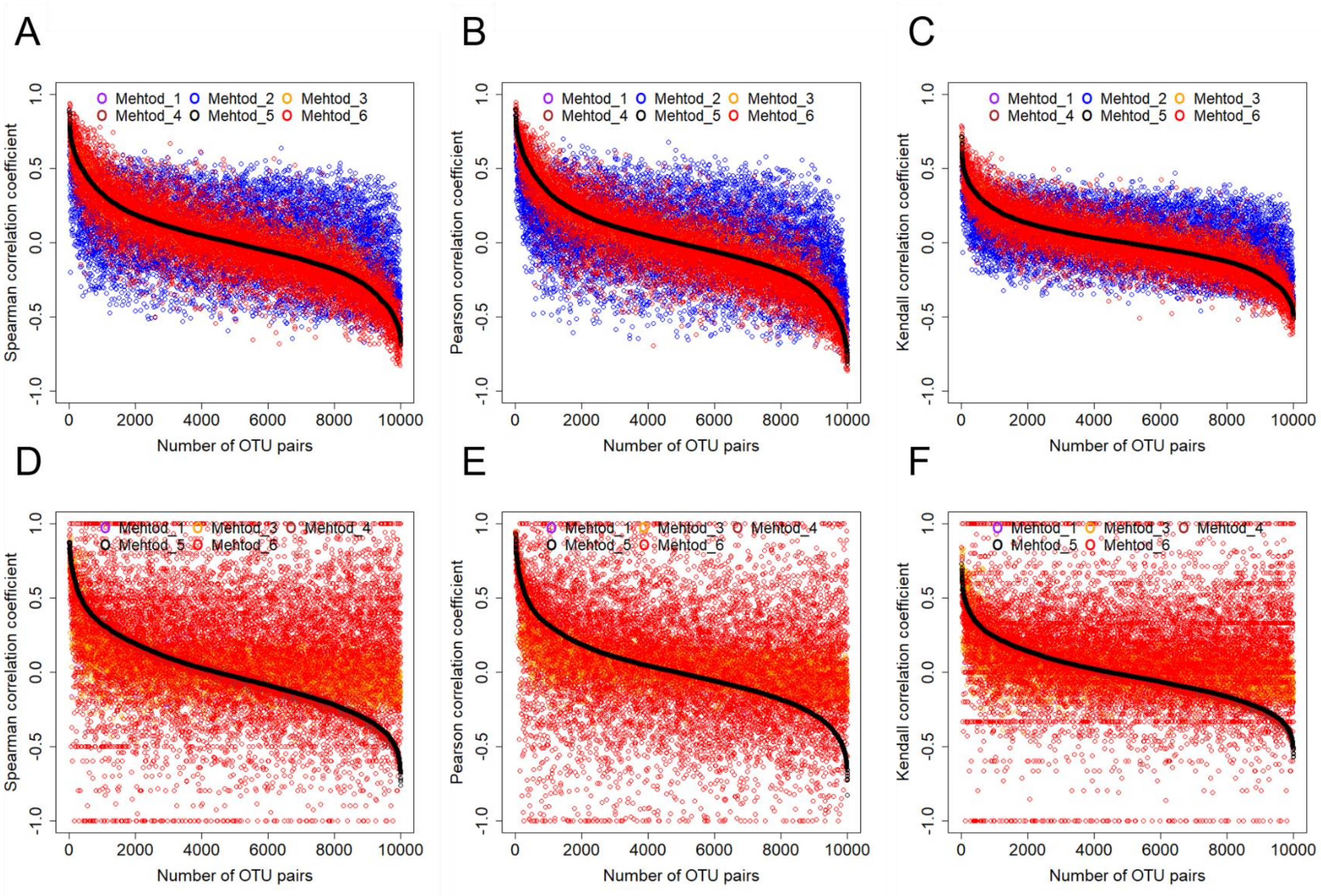

Supplementary Figure 2. The effects of data filtering methods on correlation inference using centered log transformed microbial profiles. Two types of datasets, including OTUs showing up in ≥ 60 samples (A-C) and OTUs showing up in ≥ 30 samples and ≤ 60 samples (D-F) were analyzed. Three different correlation calculation methods were evaluated, including Spearman, Pearson, and Kendall. For better visualization, correlation coefficient values of 10 000 randomly selected OTU pairs were plotted. For the less frequent dataset (D-F), nearest neighbor algorithm was not applicable.

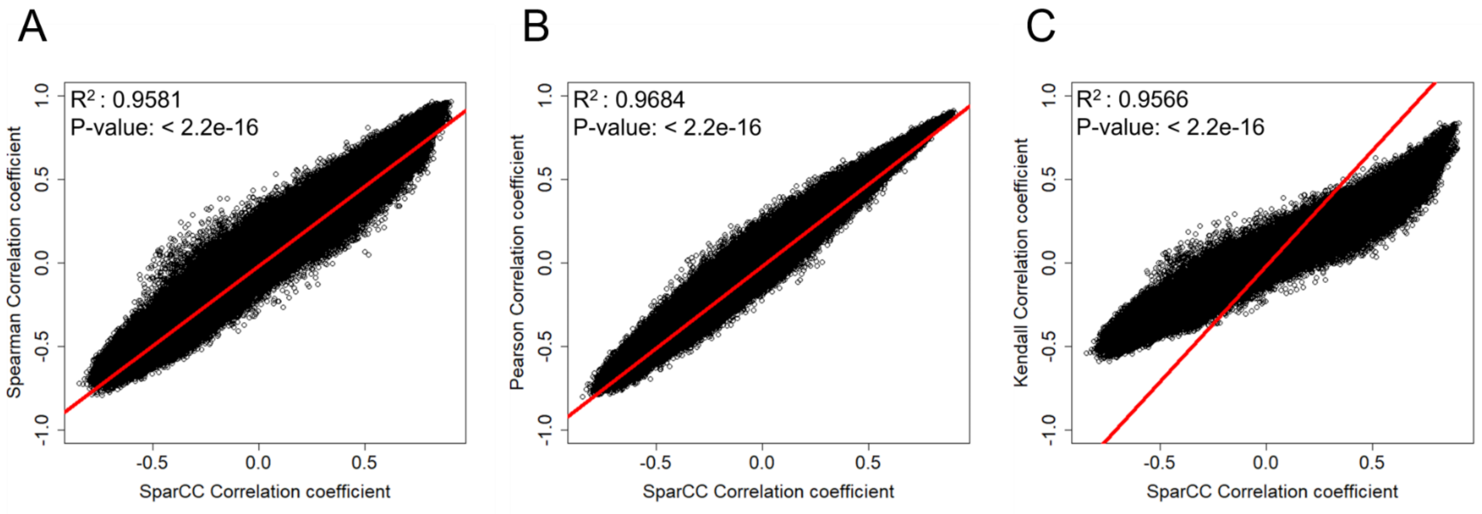

Supplementary Figure 3. Comparison of the correlation coefficient values calculated by Spearman (A), Pearson (B), and Kendall (C) correlation methods with that by SparCC among OTUs showing up in ≥ 60 samples.

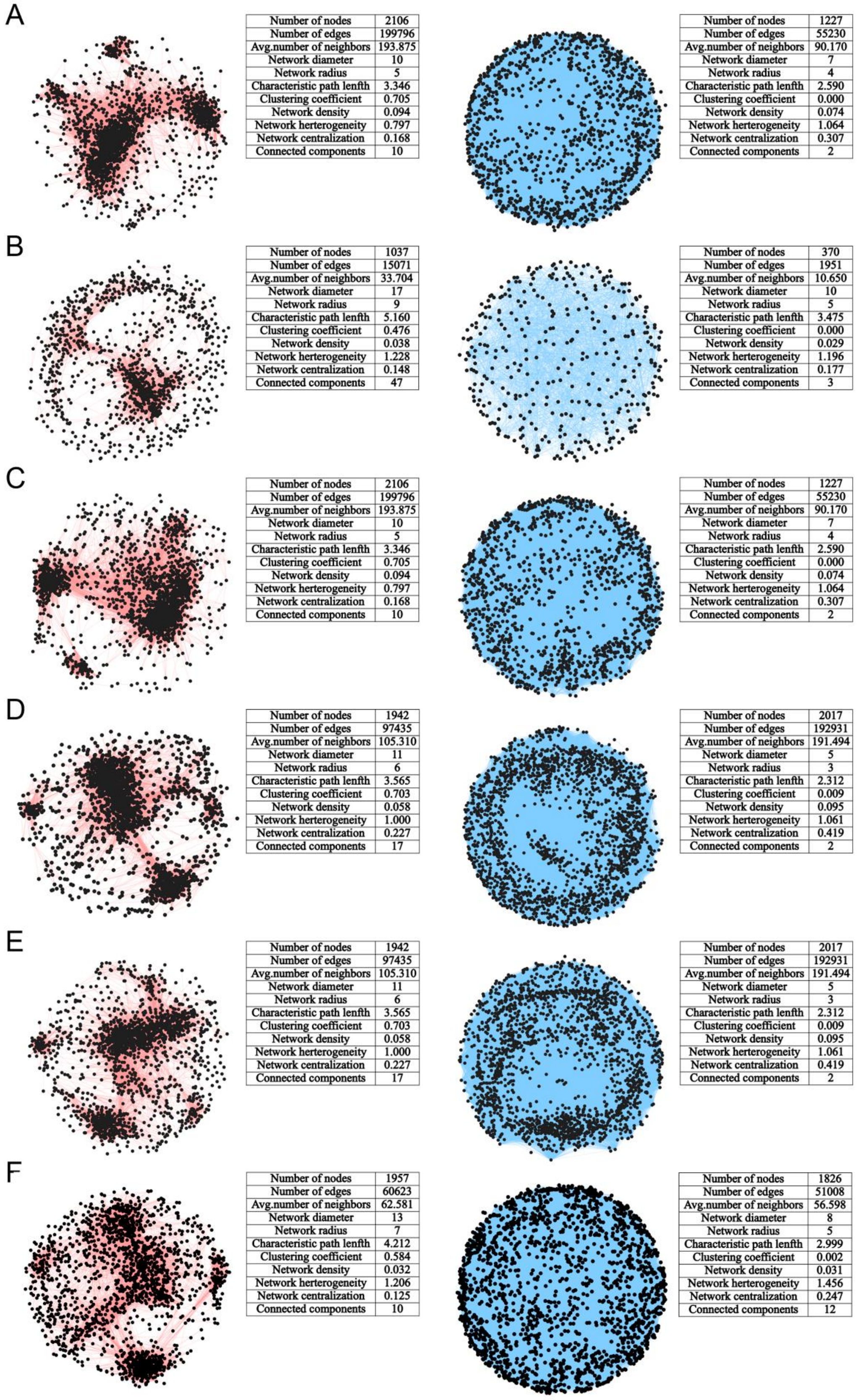

A

Number of nodes	2106	Number of nodes	1227
Number of edges	199796	Number of edges	55230
Avg.number of neighbors	193.875	Avg.number of neighbors	90.170
Network diameter	10	Network diameter	7
Network radius	5	Network radius	4
Characteristic path lenfth	3.346	Characteristic path lenfth	2.590
Clustering coefficient	0.705	Clustering coefficient	0.000
Network density	0.094	Network density	0.074
Network herterogeneity	0.797	Network herterogeneity	1.064
Network centralization	0.168	Network centralization	0.307
Connected components	10	Connected components	2

B

Number of nodes	1037	Number of nodes	370
Number of edges	15071	Number of edges	1951
Avg.number of neighbors	33.704	Avg.number of neighbors	10.650
Network diameter	17	Network diameter	10
Network radius	9	Network radius	5
Characteristic path lenfth	5.160	Characteristic path lenfth	3.475
Clustering coefficient	0.476	Clustering coefficient	0.000
Network density	0.038	Network density	0.029
Network herterogeneity	1.228	Network herterogeneity	1.196
Network centralization	0.148	Network centralization	0.177
Connected components	47	Connected components	3

C

Number of nodes	2106	Number of nodes	1227
Number of edges	199796	Number of edges	55230
Avg.number of neighbors	193.875	Avg.number of neighbors	90.170
Network diameter	10	Network diameter	7
Network radius	5	Network radius	4
Characteristic path lenfth	3.346	Characteristic path lenfth	2.590
Clustering coefficient	0.705	Clustering coefficient	0.000
Network density	0.094	Network density	0.074
Network herterogeneity	0.797	Network herterogeneity	1.064
Network centralization	0.168	Network centralization	0.307
Connected components	10	Connected components	2

D

Number of nodes	1942	Number of nodes	2017
Number of edges	97435	Number of edges	192931
Avg.number of neighbors	105.310	Avg.number of neighbors	191.494
Network diameter	11	Network diameter	5
Network radius	6	Network radius	3
Characteristic path lenfth	3.565	Characteristic path lenfth	2.312
Clustering coefficient	0.703	Clustering coefficient	0.009
Network density	0.058	Network density	0.095
Network herterogeneity	1.000	Network herterogeneity	1.061
Network centralization	0.227	Network centralization	0.419
Connected components	17	Connected components	2

E

Number of nodes	1942	Number of nodes	2017
Number of edges	97435	Number of edges	192931
Avg.number of neighbors	105.310	Avg.number of neighbors	191.494
Network diameter	11	Network diameter	5
Network radius	6	Network radius	3
Characteristic path lenfth	3.565	Characteristic path lenfth	2.312
Clustering coefficient	0.703	Clustering coefficient	0.009
Network density	0.058	Network density	0.095
Network herterogeneity	1.000	Network herterogeneity	1.061
Network centralization	0.227	Network centralization	0.419
Connected components	17	Connected components	2

F

Number of nodes	1957	Number of nodes	1826
Number of edges	60623	Number of edges	51008
Avg.number of neighbors	62.581	Avg.number of neighbors	56.598
Network diameter	13	Network diameter	8
Network radius	7	Network radius	5
Characteristic path lenfth	4.212	Characteristic path lenfth	2.999
Clustering coefficient	0.584	Clustering coefficient	0.002
Network density	0.032	Network density	0.031
Network herterogeneity	1.206	Network herterogeneity	1.456
Network centralization	0.125	Network centralization	0.247
Connected components	10	Connected components	12

Supplementary Figure 4. The microbial co-occurrence and co-exclusive networks were based on Spearman correlation coefficient, using different data filtering methods with OTUs showing up in ≥ 60 samples. Correlation coefficient cutoffs are 0.6 and -0.6 ($P < 0.001$) for positive and negative associations, respectively. Networks with red edges represent positive correlation, and networks with blue edges represent negative correlation. All six different data filtering methods were analyzed: (A) The first data filtering method; (B) The second data filtering method; (C) The third data filtering method; (D) The fourth data filtering method; (E) The fifth data filtering method; (F) The sixth data filtering method.

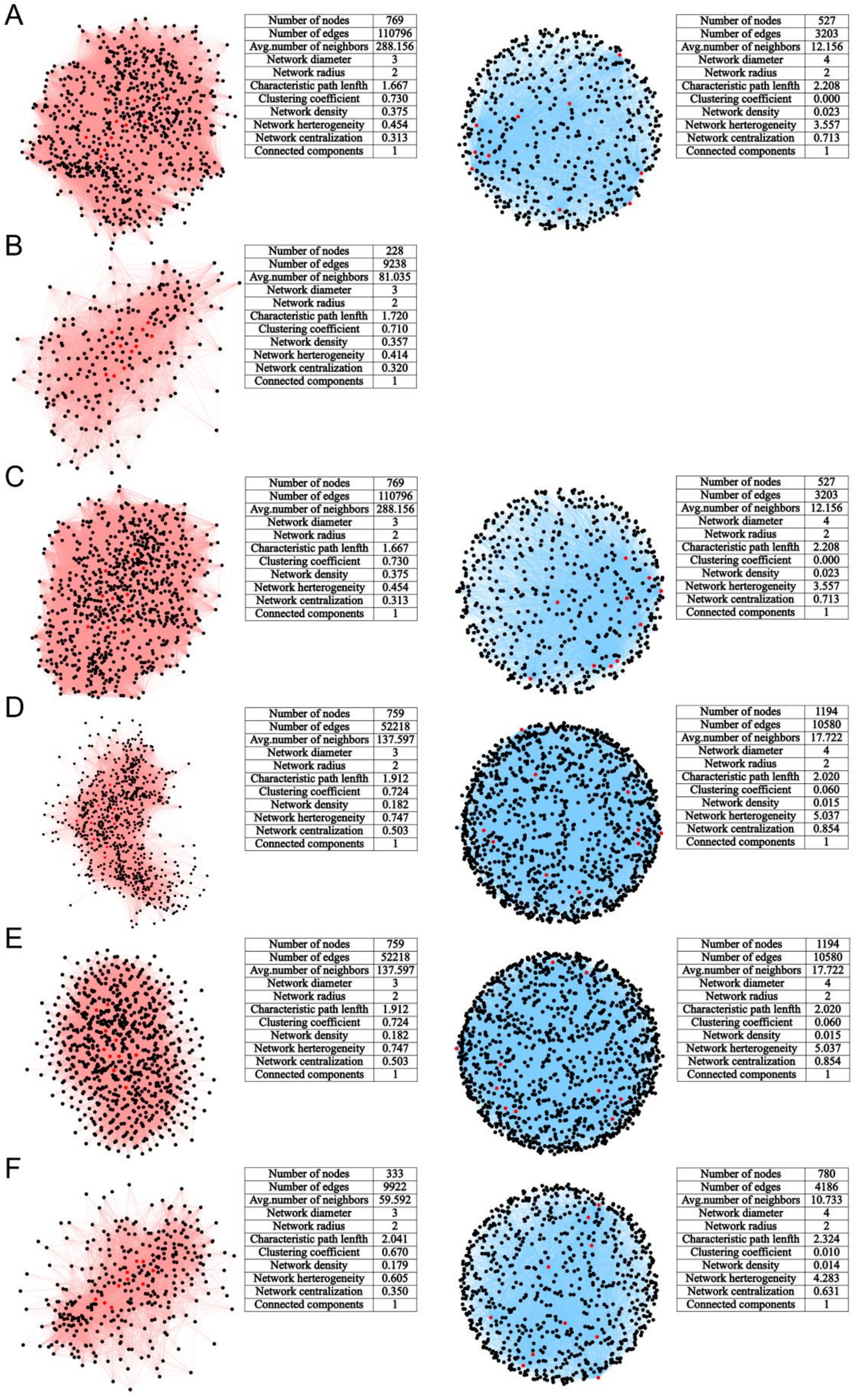

A

Number of nodes	769
Number of edges	110796
Avg.number of neighbors	288.156
Network diameter	3
Network radius	2
Characteristic path lenfth	1.667
Clustering coefficient	0.730
Network density	0.375
Network herterogeneity	0.454
Network centralization	0.313
Connected components	1

Number of nodes	527
Number of edges	3203
Avg.number of neighbors	12.156
Network diameter	4
Network radius	2
Characteristic path lenfth	2.208
Clustering coefficient	0.000
Network density	0.023
Network herterogeneity	3.557
Network centralization	0.713
Connected components	1

B

Number of nodes	228
Number of edges	9238
Avg.number of neighbors	81.035
Network diameter	3
Network radius	2
Characteristic path lenfth	1.720
Clustering coefficient	0.710
Network density	0.357
Network herterogeneity	0.414
Network centralization	0.320
Connected components	1

C

Number of nodes	769
Number of edges	110796
Avg.number of neighbors	288.156
Network diameter	3
Network radius	2
Characteristic path lenfth	1.667
Clustering coefficient	0.730
Network density	0.375
Network herterogeneity	0.454
Network centralization	0.313
Connected components	1

Number of nodes	527
Number of edges	3203
Avg.number of neighbors	12.156
Network diameter	4
Network radius	2
Characteristic path lenfth	2.208
Clustering coefficient	0.000
Network density	0.023
Network herterogeneity	3.557
Network centralization	0.713
Connected components	1

D

Number of nodes	759
Number of edges	52218
Avg.number of neighbors	137.597
Network diameter	3
Network radius	2
Characteristic path lenfth	1.912
Clustering coefficient	0.724
Network density	0.182
Network herterogeneity	0.747
Network centralization	0.503
Connected components	1

Number of nodes	1194
Number of edges	10580
Avg.number of neighbors	17.722
Network diameter	4
Network radius	2
Characteristic path lenfth	2.020
Clustering coefficient	0.060
Network density	0.015
Network herterogeneity	5.037
Network centralization	0.854
Connected components	1

E

Number of nodes	759
Number of edges	52218
Avg.number of neighbors	137.597
Network diameter	3
Network radius	2
Characteristic path lenfth	1.912
Clustering coefficient	0.724
Network density	0.182
Network herterogeneity	0.747
Network centralization	0.503
Connected components	1

Number of nodes	1194
Number of edges	10580
Avg.number of neighbors	17.722
Network diameter	4
Network radius	2
Characteristic path lenfth	2.020
Clustering coefficient	0.060
Network density	0.015
Network herterogeneity	5.037
Network centralization	0.854
Connected components	1

F

Number of nodes	333
Number of edges	9922
Avg.number of neighbors	59.592
Network diameter	3
Network radius	2
Characteristic path lenfth	2.041
Clustering coefficient	0.670
Network density	0.179
Network herterogeneity	0.605
Network centralization	0.350
Connected components	1

Number of nodes	780
Number of edges	4186
Avg.number of neighbors	10.733
Network diameter	4
Network radius	2
Characteristic path lenfth	2.324
Clustering coefficient	0.010
Network density	0.014
Network herterogeneity	4.283
Network centralization	0.631
Connected components	1

Supplementary Figure 5. The microbial co-occurrence and co-exclusive subnetworks of first neighbors of top ten most connected nodes were identified in the fifth data filtering method based on Spearman correlation coefficient, using different data filtering methods with OTUs showing up in ≥ 60 samples. Correlation coefficient cutoffs are 0.6 and -0.6 ($P < 0.001$) for positive and negative associations, respectively. Networks with red edges represent positive correlation, and networks with blue edges represent negative correlation. All six different data filtering methods were analyzed: (A) The first data filtering method; (B) The second data filtering method, but top ten most connected nodes identified in the fifth data filtering method cannot be found in co-exclusive networks; (C) The third data filtering method; (D) The fourth data filtering method; (E) The fifth data filtering method; (F) The sixth data filtering method.

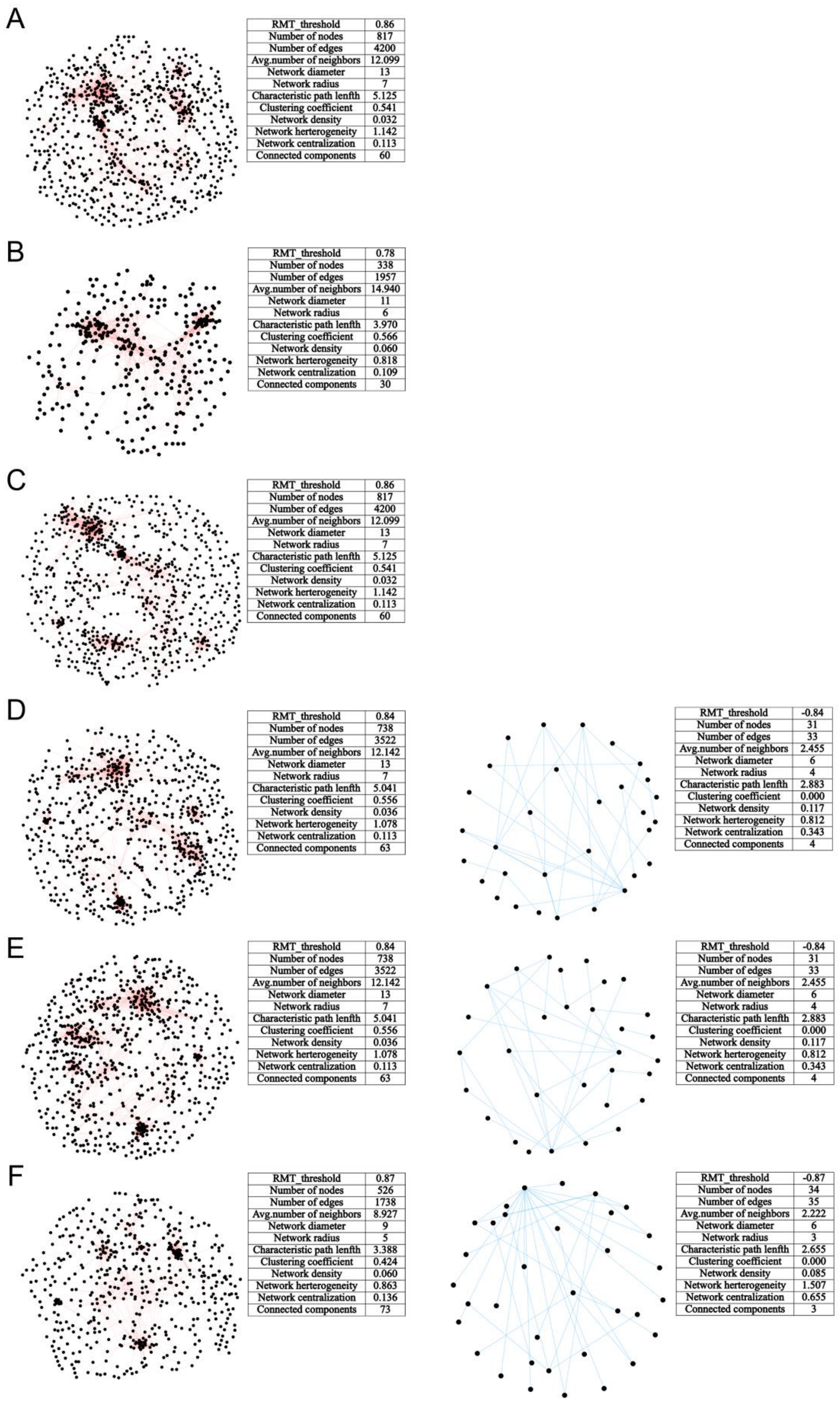

A

RMT_threshold	0.86
Number of nodes	817
Number of edges	4200
Avg.number of neighbors	12.099
Network diameter	13
Network radius	7
Characteristic path lenfth	5.125
Clustering coefficient	0.541
Network density	0.032
Network herterogeneity	1.142
Network centralization	0.113
Connected components	60

B

RMT_threshold	0.78
Number of nodes	338
Number of edges	1957
Avg.number of neighbors	14.940
Network diameter	11
Network radius	6
Characteristic path lenfth	3.970
Clustering coefficient	0.566
Network density	0.060
Network herterogeneity	0.818
Network centralization	0.109
Connected components	30

C

RMT_threshold	0.86
Number of nodes	817
Number of edges	4200
Avg.number of neighbors	12.099
Network diameter	13
Network radius	7
Characteristic path lenfth	5.125
Clustering coefficient	0.541
Network density	0.032
Network herterogeneity	1.142
Network centralization	0.113
Connected components	60

D

RMT_threshold	0.84
Number of nodes	738
Number of edges	3522
Avg.number of neighbors	12.142
Network diameter	13
Network radius	7
Characteristic path lenfth	5.041
Clustering coefficient	0.556
Network density	0.036
Network herterogeneity	1.078
Network centralization	0.113
Connected components	63

RMT_threshold	-0.84
Number of nodes	31
Number of edges	33
Avg.number of neighbors	2.455
Network diameter	6
Network radius	4
Characteristic path lenfth	2.883
Clustering coefficient	0.000
Network density	0.117
Network herterogeneity	0.812
Network centralization	0.343
Connected components	4

E

RMT_threshold	0.84
Number of nodes	738
Number of edges	3522
Avg.number of neighbors	12.142
Network diameter	13
Network radius	7
Characteristic path lenfth	5.041
Clustering coefficient	0.556
Network density	0.036
Network herterogeneity	1.078
Network centralization	0.113
Connected components	63

RMT_threshold	-0.84
Number of nodes	31
Number of edges	33
Avg.number of neighbors	2.455
Network diameter	6
Network radius	4
Characteristic path lenfth	2.883
Clustering coefficient	0.000
Network density	0.117
Network herterogeneity	0.812
Network centralization	0.343
Connected components	4

F

RMT_threshold	0.87
Number of nodes	526
Number of edges	1738
Avg.number of neighbors	8.927
Network diameter	9
Network radius	5
Characteristic path lenfth	3.388
Clustering coefficient	0.424
Network density	0.060
Network herterogeneity	0.863
Network centralization	0.136
Connected components	73

RMT_threshold	-0.87
Number of nodes	34
Number of edges	35
Avg.number of neighbors	2.222
Network diameter	6
Network radius	3
Characteristic path lenfth	2.655
Clustering coefficient	0.000
Network density	0.085
Network herterogeneity	1.507
Network centralization	0.655
Connected components	3

Supplementary Figure 6. The microbial co-occurrence and co-exclusive networks were based on Spearman correlation coefficient, using different data filtering methods with OTUs showing up in ≥ 60 samples. The RMT method was used to determine correlation coefficient cutoffs via the iNAP pipeline. A P-value cutoff of 0.001 was also applied for network construction. Networks with red edges represent positive correlation, and networks with blue edges represent negative correlation. All six different data filtering methods were analyzed: (A) The first data filtering method; (B) The second data filtering method; (C) The third data filtering method; (D) The fourth data filtering method; (E) The fifth data filtering method; (F) The sixth data filtering method.

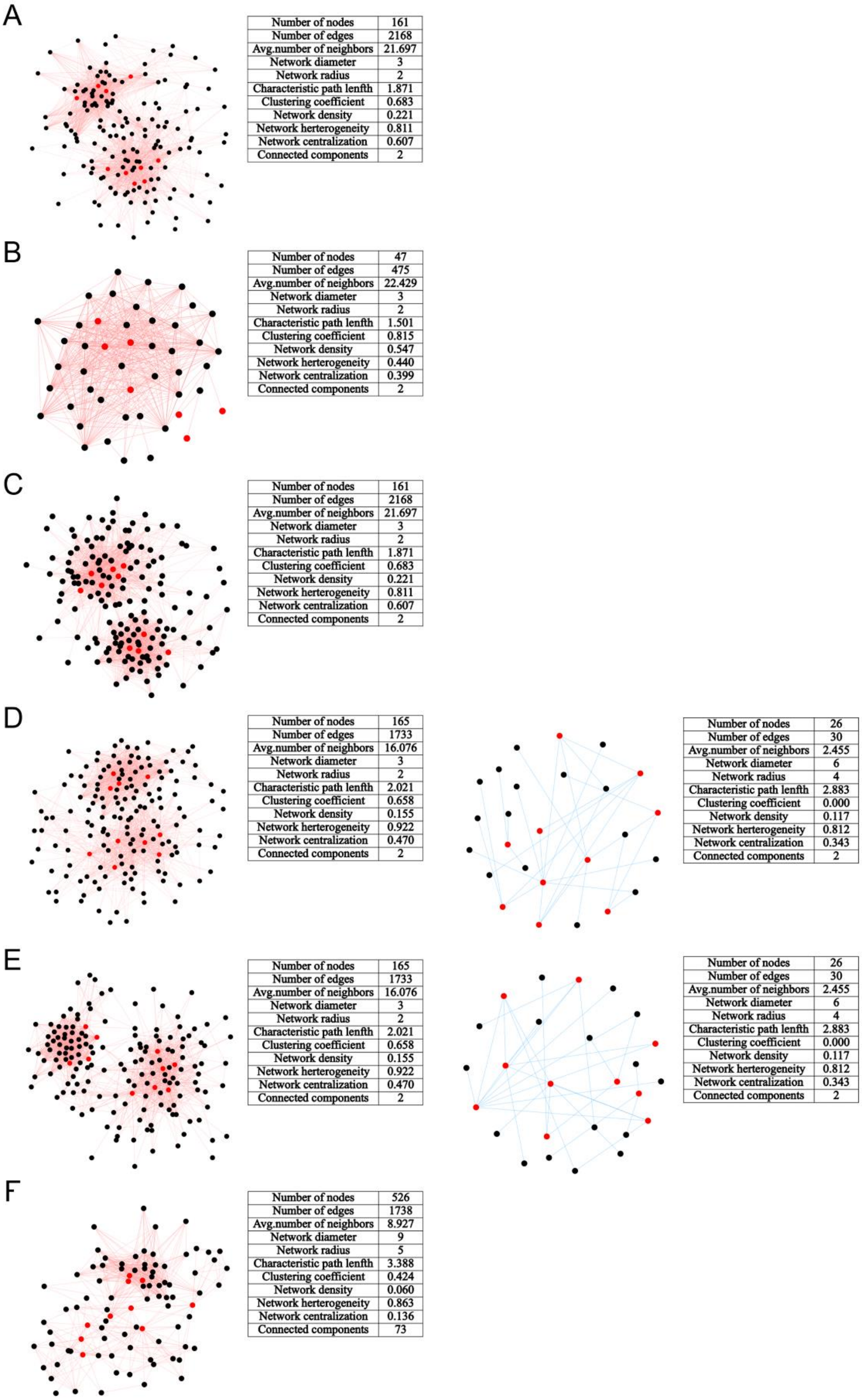

A

Number of nodes	161
Number of edges	2168
Avg.number of neighbors	21.697
Network diameter	3
Network radius	2
Characteristic path lenfth	1.871
Clustering coefficient	0.683
Network density	0.221
Network herterogeneity	0.811
Network centralization	0.607
Connected components	2

B

Number of nodes	47
Number of edges	475
Avg.number of neighbors	22.429
Network diameter	3
Network radius	2
Characteristic path lenfth	1.501
Clustering coefficient	0.815
Network density	0.547
Network herterogeneity	0.440
Network centralization	0.399
Connected components	2

C

Number of nodes	161
Number of edges	2168
Avg.number of neighbors	21.697
Network diameter	3
Network radius	2
Characteristic path lenfth	1.871
Clustering coefficient	0.683
Network density	0.221
Network herterogeneity	0.811
Network centralization	0.607
Connected components	2

D

Number of nodes	165
Number of edges	1733
Avg.number of neighbors	16.076
Network diameter	3
Network radius	2
Characteristic path lenfth	2.021
Clustering coefficient	0.658
Network density	0.155
Network herterogeneity	0.922
Network centralization	0.470
Connected components	2

Number of nodes	26
Number of edges	30
Avg.number of neighbors	2.455
Network diameter	6
Network radius	4
Characteristic path lenfth	2.883
Clustering coefficient	0.000
Network density	0.117
Network herterogeneity	0.812
Network centralization	0.343
Connected components	2

E

Number of nodes	165
Number of edges	1733
Avg.number of neighbors	16.076
Network diameter	3
Network radius	2
Characteristic path lenfth	2.021
Clustering coefficient	0.658
Network density	0.155
Network herterogeneity	0.922
Network centralization	0.470
Connected components	2

Number of nodes	26
Number of edges	30
Avg.number of neighbors	2.455
Network diameter	6
Network radius	4
Characteristic path lenfth	2.883
Clustering coefficient	0.000
Network density	0.117
Network herterogeneity	0.812
Network centralization	0.343
Connected components	2

F

Number of nodes	526
Number of edges	1738
Avg.number of neighbors	8.927
Network diameter	9
Network radius	5
Characteristic path lenfth	3.388
Clustering coefficient	0.424
Network density	0.060
Network herterogeneity	0.863
Network centralization	0.136
Connected components	73

Supplementary Figure 7. The microbial co-occurrence and co-exclusive subnetworks of first neighbors of top ten most connected nodes were identified in the fifth data filtering method based on Spearman correlation coefficient, using different data filtering methods with OTUs showing up in ≥ 60 samples. The RMT method was used to determine correlation coefficient cutoffs via the iNAP pipeline. A P-value cutoff of 0.001 was also applied for network construction. Networks with red edges represent positive correlation, and networks with blue edges represent negative correlation. All six different data filtering methods were analyzed: (A) The first data filtering method; (B) The second data filtering method; (C) The third data filtering method; (D) The fourth data filtering method; (E) The fifth data filtering method; (F) The sixth data filtering method.

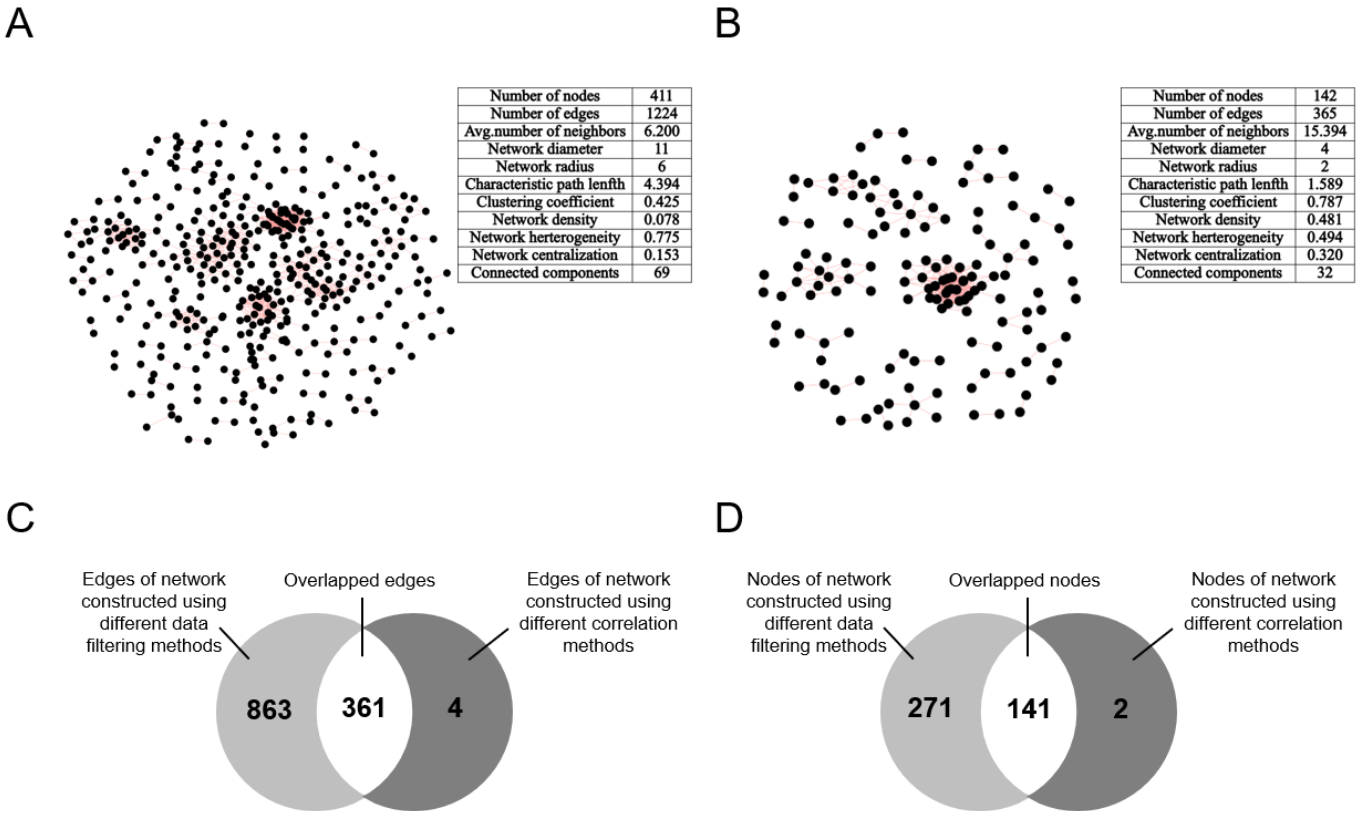

Supplementary Figure 8. Consensus networks were constructed and comparatively analyzed. The iNAP pipeline was applied to determine the cutoffs for network construction based on RMT method. A P-value cutoff of 0.001 was also applied for network construction. Consensus co-exclusive networks were not found due to the high cutoff of RMT approach. (A) Consensus co-occurrence networks were constructed by extracting the overlapped nodes and edges based on networks constructed using the first, third, fifth, and sixth data filtering methods (refer to Fig. 1). Spearman's rank order correlation coefficient was used for network construction. (B) Consensus co-occurrence networks were constructed by extracting the overlapped nodes and edges based on networks constructed using different correlation methods, including Spearman, Pearson, Kendall, and SparCC. The first data filtering method (filling missing values with zero) was used here. (C) Overlapped edges between the consensus networks of different data filtering methods and correlation methods. (D) Overlapped nodes between the consensus networks of different data filtering methods and correlation methods.
